# Correction to ‘The Relationship Between Attachment Styles and Love Attitudes of Undergraduate Nursing Students’

**DOI:** 10.1002/nop2.70414

**Published:** 2026-01-30

Ozeren G S. 2025. “The Relationship Between Attachment Styles and Love Attitudes of Undergraduate Nursing Students.” *Nursing Open* 12, no. 10: e70318. https://doi.org/10.1002/nop2.70318.

Two instances of de‐blinding were not resolved prior to initial publication. These have both now been fixed within the current version of the article, and are as follows:

In the ‘Design and Methods’ section of the abstract, the text ‘This study was conducted at a state university in northern X’ has been corrected to ‘This study was conducted at a state university in northern Turkiye’. In section 2.5 ‘Ethical Principles’, the text ‘Permission was obtained from the Human Research Ethics Committee of X University prior to the commencement of the research (2019–2045)’ has been corrected to ‘Permission was obtained from the Human Research Ethics Committee of the Sinop University prior to the commencement of the research (2019‐45)’.

We apologize for this error.

